# Excessive Substitution of Fish Meal with Fermented Soybean Meal Induces Oxidative Stress by Impairing Glutathione Metabolism in Largemouth Bass (*Micropterus salmoides*)

**DOI:** 10.3390/antiox12122096

**Published:** 2023-12-11

**Authors:** Qiang Chen, Congcong Wang, Yulong Sun, Yan Chen, Songming Chen, Tao Han, Jiteng Wang

**Affiliations:** 1 Department of Aquaculture, Zhejiang Ocean University, Zhoushan 316022, China; chenqiang@zjou.edu.cn (Q.C.);; 2 Zhejiang Provincial Engineering Technology Research Center of Marine Biomedical Products, School of Food and Pharmacy, Zhejiang Ocean University, Zhoushan 316022, China

**Keywords:** fermented soybean meal, glycine, glutathione metabolism, *Micropterus salmoides*, oxidative stress

## Abstract

The application of fermented soybean meal (FSBM) is an effective strategy to alleviate the shortage of fish meal (FM) in aquaculture. However, an excessive substitution ratio often reduces fish growth and induces liver oxidative stress, while the mechanism remains poorly understood. Here, an 8-week feeding trial was conducted in largemouth bass (initial weight: 6.82 ± 0.09 g) to establish an oxidative stress model by replacing 50% of FM with FSBM (fermented by *Bacillus subtilis*). The results showed that FSBM substitution significantly reduced the growth performance of largemouth bass, including the weight gain rate and specific growth rate. Moreover, FSBM significantly reduced the contents of essential amino acids and total free amino acids in muscle, along with the mRNA expression of amino acids and small peptide transporters. Enzyme activity detection and liver sections showed that FSBM substitution caused liver oxidative stress, indicating the successful construction of an oxidative stress model. An integrated analysis of transcriptomic and metabolomic data revealed that FSBM substitution impaired glycine, serine and threonine metabolism, as well as glutathione metabolism. In addition, the ratio of reduced glutathione (GSH) to oxidized glutathione (GSSG) was decreased in the FSBM group, which may explain the mechanism of oxidative stress caused by FSBM substitution. Considering that glycine is an important component of glutathione synthesis, key genes involved in glycine metabolism (*glya*, *gnmt* and *agxt*) and dietary glycine supplementation should be valued to improve the availability of FSBM. This study reveals for the first time the importance of non-essential amino acids in improving the utilization of plant-based protein sources and provides original insight for the optimization of aquatic feeds.

## 1. Introduction

Finding a suitable protein source to replace fish meal (FM) is a crucial method to ensure the healthy and sustainable growth of aquaculture, particularly given the scarcity of FM supplies and the increase their price. Vegetable protein source soybean meal (SBM) is a viable alternative to FM because of its inexpensive cost and plentiful availability [1]. Moreover, SBM is improved via fermentation technology to decrease anti-nutritional factor (ANF) levels and increase crude protein contents [2,3]. Fermented soybean meal (FSBM) is widely applied in aquaculture [4,5]. However, the ability of FSBM to completely replace FM in aquatic feed is currently restricted, and excessive FSBM substitution reduces the growth performance of fish and induces liver damage [6].

The growth-promoting function of protein sources in feed depends on the amino acid profile. Hence, in addition to the inevitable ANFs, the restriction of FSBM substitution is largely due to its unbalanced amino acid composition [7,8]. In the past, more attention has been placed on the differences in essential amino acid contents between FSBM and FM, including methionine (Met), lysine (Lys) and threonine (Thr). Correspondingly, crystalline amino acids were also added to feed to improve the utilization of FSBM by aquatic animals [9,10]. In contrast, non-essential amino acids are less recognized. Recently, the concept of “functional amino acids” has strengthened the understanding of non-essential amino acid functions [11]. In addition to being protein substrates, they also have vital biological functions such as regulating metabolism and improving immunity [12]. Among them, the content of glycine (Gly) in FSBM is lower than that in FM as shown in previous findings in our lab and a published study [13]. Gly promotes muscle development and relieves inflammatory responses [14,15]. Moreover, as a synthetic substance of glutathione [16], Gly enhances the antioxidant capacity both in mammals and aquatic animals [17,18]. Hence, excessive FSBM may cause oxidative stress by disrupting Gly-glutathione metabolism, thereby reducing the growth of fish. However, the specific mechanism still requires further exploration.

Largemouth bass (*Micropterus salmoides*) is one of the most important freshwater cultured species in China, with a total production of about 700,000 tons (China Fishery Statistical Yearbook, 2022). However, as a carnivorous fish, largemouth bass has a high requirement for dietary protein, which mainly comes from FM [19]. It is of great economic value to investigate the parameters that restrict the substitution of FM with FSBM. The data from our lab and previous studies indicated that FSBM could replace 30–40% of FM in feed without negative effects on the growth performance and liver health of largemouth bass [20,21]. In the present study, soybean meal was fermented using *Bacillus subtilis* to improve its nutritional value. An 8-week feeding trial was conducted to explore the mechanism of growth inhibition and oxidative stress caused by excessive substitution of FM with FSBM (replacing 50% of FM with FSBM). Through amino acid analysis, histology and qRT-PCR, combined with transcriptomics and metabolomics analyses, we found that excessive FSBM contributes to oxidative stress and liver injury by disrupting glycine-serine-threonine metabolism and glutathione metabolism in largemouth bass, which is an important reason to limit the proportion of FSBM replacement. Due to the relationship between Gly and glutathione, these findings highlight the importance of non-essential amino acids and may provide theoretical guidance for the improvement of aquatic feed formula.

## 2. Materials and Methods

### 2.1. Ethics Statement

In the current study, experimental fish were handled and utilized in compliance with the animal welfare protocols authorized by Zhejiang Ocean University, as well as local and international guidelines.

### 2.2. Experimental Diets

Soybean meal was fermented using *Bacillus subtilis* according to a previously described method [22]. The amino acid profile of FSBM is shown in Supplementary Table S1. Two isonitrogenous (approximately 46% crude protein) and isolipidic (approximately 12% crude lipid) diets were formulated. The control diet (CON) contained 62% FM, while in the FSBM diet, 50% of FM was substituted with FSBM. Based on the protein requirement [23] and the characteristic of high dietary protein demand [19], the crude protein was set to about 48%. Crystalline Met and Lys were supplemented in the FSBM diet to satisfy the growth needs of largemouth bass [24,25]. Correspondingly, alanine was used to maintain a nitrogen balance in the CON diet. Details on feed formulations are provided in Table 1. After smashing, mixing and extruded pelletizing, all the pellets were stored at −20 °C until use. The amino acid composition of the experimental diets is shown in Table 2.

### 2.3. Animal Feeding Procedure and Sampling

Juvenile largemouth bass were purchased from Chia Tai Aquatic Products Co., Ltd. (Huzhou, Zhejiang, China). After acclimating to the experimental conditions for two weeks, 120 healthy fish with similar sizes (mean weight 6.82 ± 0.09 g) were randomly divided into 6 tanks (water volume, 150 L), with triplicate groups for each diet. Fish were fed to apparent satiation twice daily at 8:00 and 17:00 for 8 weeks. The water conditions (temperature: 26 ± 2 °C; dissolved oxygen: 6–7 mg/L) were optimal for largemouth bass growth during the experimental period.

At the end of the experiment, fish were starved for 24 h and anesthetized with MS-222 (1:10,000; Sigma, St. Louis, MO, USA). All the fish were weighed to determine the weight gain rate (WG), specific growth rate (SGR) and feed efficiency ratio (FER). The weights of liver and the visceral mass were recorded to calculate the hepato-somatic index (HSI) and the viscera-somatic index (VSI). Six fish from each tank were utilized to collect serum, liver and muscle samples, which were then stored at −80 °C until analyzed.

### 2.4. Biochemical and Histological Analysis

The moisture of feed was assessed in a lyophilizer (Freeze Dryer LL1500, Thermo Fisher Scientific, Waltham, MA, USA) by drying to a consistent weight. The crude lipids in the feed were measured with the ether extraction method using Soxhlet apparatus (E816, Buchi, Flawil, Switzerland). The crude protein concentration was analyzed via the Kjeldahl method (N × 6.25) (K355/K437, Buchi, Flawil, Switzerland). Ash was measured with a muffle furnace at 550 °C for 12 h. All the above methods complied with the details described by the Association of Official Analytical Chemists (AOAC) [27].

The antioxidant indexes in the serum and liver were determined with commercially available kits (Nanjing Jiancheng Bioengineering Inc., Nanjing, China). The available kit information is listed as follows: alanine transaminase (ALT) assay kit (cat. no. C009-2-1), aspartate aminotransferase (AST) assay kit (cat. no. C010-2-1), superoxide dismutase (SOD) assay kit (WST-1 method) (cat. no. A001-3-2), catalase (CAT) assay kit (Visible light) (cat. no. A007-1-1), total antioxidant capacity (T-AOC) assay kit (ABTS method) (cat. no. A015-2-1) and reduced glutathione (GSH) assay kit (cat. no. A006-2-1).

Pieces of livers were fixed in 4% paraformaldehyde for histochemical and histological examination. For morphological investigations, paraffin-embedded liver slices were stained with hematoxylin and eosin (H&E).

### 2.5. Free Amino Acid Analyses in Serum and Muscle

The free amino acid profiles in serum and muscle were measured with a lipid chromatograph (Agilent, 1260 Infinity Ⅱ, Santa Clara, CA, USA) according to the manufacturer’s instructions. Briefly, salicylic acid was used to hydrolyze muscle homogenate or serum. Acetonitrile and sodium bicarbonate were then added and mixed at a ratio of 1:1. After centrifuging the mixture for 10 min at 15,000 rpm, the supernatant was measured using a lipid chromatograph.

### 2.6. RNA Extraction, Reverse Transcription, and Real-Time Polymerase Chain Reaction (qPCR)

The total RNA of liver and muscle was extracted with TRIzol Reagent (R401-01, Vazyme, Nanjing, China) according to a previously described method [28]. After quantification by a NanoDrop 2000 spectrophotometer (Thermo Fisher Scientific, Waltham, MA, USA), the RNA was reverse transcribed into first-strand cDNA using a PrimeScript RT Reagent Kit (RR047Q, Takara, Japan). qPCR was performed using SYBR qPCR Master Mix (Q711-02, Vazyme) following approved methods. Primers were developed in line with published NCBI sequences and are reported in Supplementary Table S2. In the trial, the mRNA expression of glyceraldehyde 3-phosphate dehydrogenase (*gapdh*) was not affected by different diets. Hence, *gapdh* was used as the housekeeping gene. The comparative cycle threshold (CT) approach (2^−ΔΔCT^ method) was used to quantify gene mRNA levels [29].

### 2.7. Transcriptomic Analysis in the Liver

A transcriptomic analysis was conducted according to a previously described method [30]. In brief, three liver samples from each diet were sent to Biomarker Technologies Co. (Beijing, China). After obtaining high-quality RNA, sequencing was performed on an Illumina NovaSeq6000. The bioinformatics analyses, which included a Kyoto Encyclopedia of Genes and Genomes (KEGG) enrichment analysis and a Gene Ontology (GO) analysis of differentially expressed genes (DEGs), were implemented using OmicShare tools (https://www.omicshare.com/) (accessed on 14 August 2022).

### 2.8. Untargeted Metabolomics in the Liver

Six liver samples were prepared and sent to Biomarker Technologies Co. (Beijing, China). Data quality evaluation, annotation analysis, and metabolite functional enrichment were all carried out. Furthermore, annotated metabolites were uploaded to BMKCloud (www.biocloud.net) (accessed on 14 August 2022) for further investigation of different metabolites (DMs). A bioinformatics analysis of DMs was performed on the OmicShare platform (https://www.omicshare.com/) (accessed on 14 August 2022).

### 2.9. Integrated Analysis of Transcriptomics and Metabolomics in the Liver

The DGs screened by transcriptomes and DMs screened by metabolomes were analyzed together on the OmicShare platform, including gene-metabolite association heatmap and KEGG mapping results. KEGG co-enrichment pathways and biological processes were screened out and the key metabolites and genes affected by diets were also identified.

### 2.10. Statistical Analysis

Except for the data from RNA-seq and the untargeted metabolomics, all the data are provided as the means ± standard deviation (SD). After analyzing for normality and homoscedasticity, comparisons between two groups were carried out using independent *t* tests in SPSS 23.0 software. Statistical significance was determined as *p* < 0.05. The number of replicates for each experiment is indicated in the figure legends.

## 3. Results

### 3.1. FSBM Substitution Reduced the Growth Performance of Juvenile Largemouth Bass

**Figure 1:**
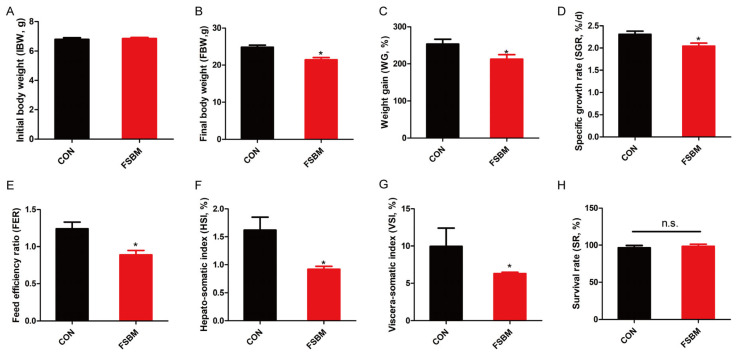
Growth parameters of largemouth bass fed the experimental diets (*n* = 3). (**A**) Initial body weight of largemouth bass. (**B**) Final body weight of largemouth bass after 8-week experiment. (**C**) Weight gain of largemouth bass after 8-week experiment. (**D**) Specific growth rate of largemouth bass after 8-week experiment. (**E**) Feed efficiency ratio of largemouth bass after 8-week experiment. (**F**) Hepato-somatic index of largemouth bass after 8-week experiment. (**G**) Viscera-somatic index of largemouth bass after 8-week experiment. (**H**) Survival rate of largemouth bass after 8-week experiment. The results are reported as means ± SD and were evaluated using independent *t*-tests (* *p* < 0.05). Weight gain rate (WG, %) = 100 × (final body wet weight-initial body wet weight)/initial body wet weight. Survival rate (SR, %) = 100 × final alive fish number/initial fish number. Specific growth rate (SGR, %/day) = (Ln (final body wet weight)—Ln (initial body wet weight)) × 100/duration of experiment (in days). Feed efficiency ratio (FER) = wet weight gain in g/dry feed fed in g. Hepato-somatic index (HIS, %) = 100 × liver wet weight/final body wet weight. Viscera-somatic index (VSI, %) = 100 × visceral wet weight/final body wet weight. N.s. means no significance.

After the 8-week feeding trial, the growth performance of juvenile largemouth bass was analyzed, as presented in Figure 1. Compared with the control diet, FSBM substitution did not affect the survival rate (Figure 1H). However, FSBM substitution significantly decreased the final body weight, weight gain rate, specific growth rate and feed efficiency ratio (Figure 1B–E; *p* < 0.05), indicating that FSBM substitution significantly inhibited the growth of largemouth bass. Moreover, FSBM substitution decreased the viscera-somatic and hepato-somatic indices (Figure 1F,G; *p* < 0.05), which demonstrated that FSBM substitution might cause liver damage.

### 3.2. FSBM Substitution Inhibited Amino Acid Transporter Expression and Decreased Total Essential Amino Acid Contents in Muscle

**Figure 2:**
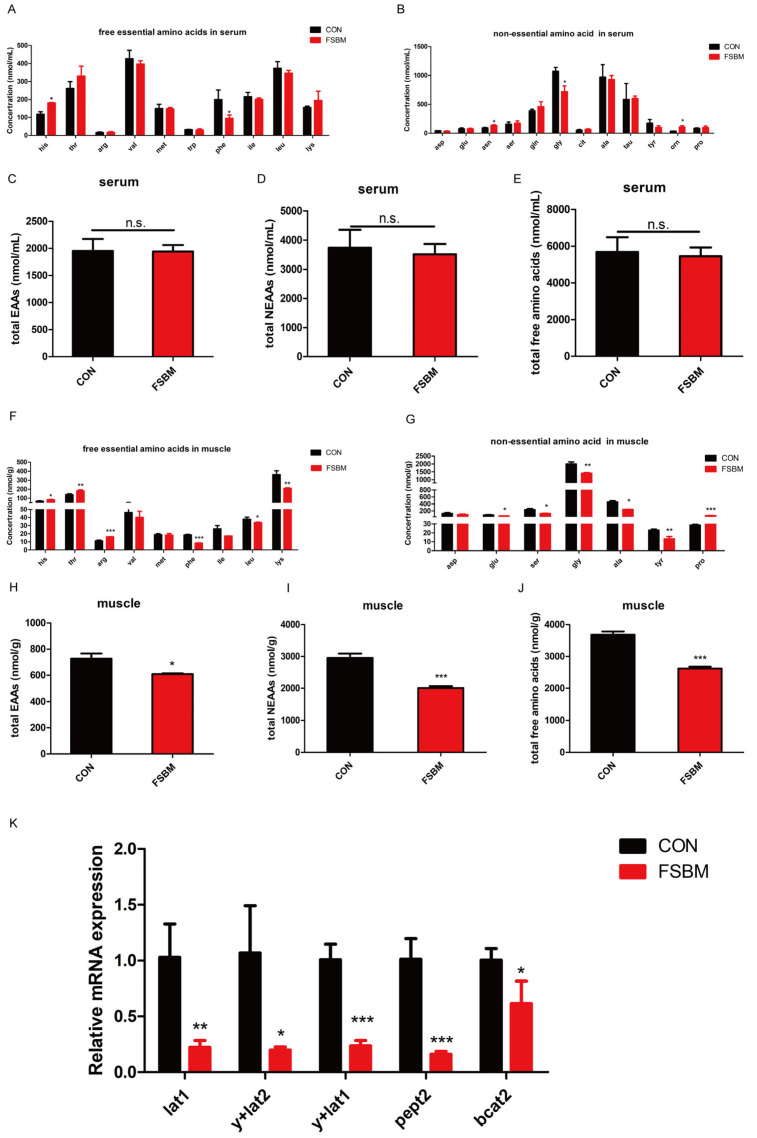
Effects of FSBM substitution on the free amino acid contents in serum (**A**–**E**) and muscle (**F**–**J**) and mRNA expression of genes associated with amino acid and small peptide transporters (**K**) in the muscle of largemouth bass (*n* = 4). The results are reported as means ± SD and were evaluated using independent *t*-tests (* *p* < 0.05, ** *p* < 0.01, *** *p* < 0.001). Arg, arginine; His, histidine; Ile, isoleucine; Leu, leucine; Lys, lysine; Met, methionine; Phe, phenylalanine; Val, valine; Thr, threonine; Trp, tryptophan; Ala, alanine; Asn, asparagine; Asp, aspartate; Cit, citrulline; Gln, glutamine; Glu, glutamate; Gly, glycine; Orn, ornithine; Pro, proline; Ser, serine; Tau, taurine; Tyr, tyrosine. EAA, essential amino acids; NEAAs, non-essential amino acids. n.s. means no significance.

The growth suppression may be due to the differences in amino acid composition between FSBM and FM, which also affects the free amino acid profile in fish. Hence, the free amino acid concentrations in serum and muscle were measured via a lipid chromatograph. Unexpectedly, FSBM substitution had little effect on free amino acids in serum. FSBM substitution significantly increased histidine (His; Figure 2A), asparagine (Asn) and ornithine (Orn) levels while decreasing phenylalanine (Phe) and glycine (Gly) levels (*p* < 0.05; Figure 2B). However, FSBM substitution had no significant influence on total essential amino acids (EAAs), total non-essential amino acids (NEAAs) and total free amino acid levels (Figure 2C–E). In contrast, FSBM substitution significantly decreased the contents of total EAAs, total NEAAs and total free amino acids in muscle (*p* < 0.05; Figure 2H–J). Further analysis revealed that FSBM substitution significantly increased His, threonine (Thr), arginine (Arg) and proline (Pro) levels, but decreased Phe, leucine (Leu), lysine (Lys), glutamate (Glu), serine (Ser), Gly, alanine (Ala) and tyrosine (Tyr) levels (*p* < 0.05; Figure 2F,G). The qRCR results showed that FSBM substitution significantly decreased the mRNA expression of amino acids, small peptide transporters (*lat1*, *y* + *lat2*, *y* + *lat1* and *pept2*) and *bcat2* in muscle (Figure 2K).

### 3.3. FSBM Substitution Caused Liver Damage by Inducing Oxidative Stress

**Figure 3:**
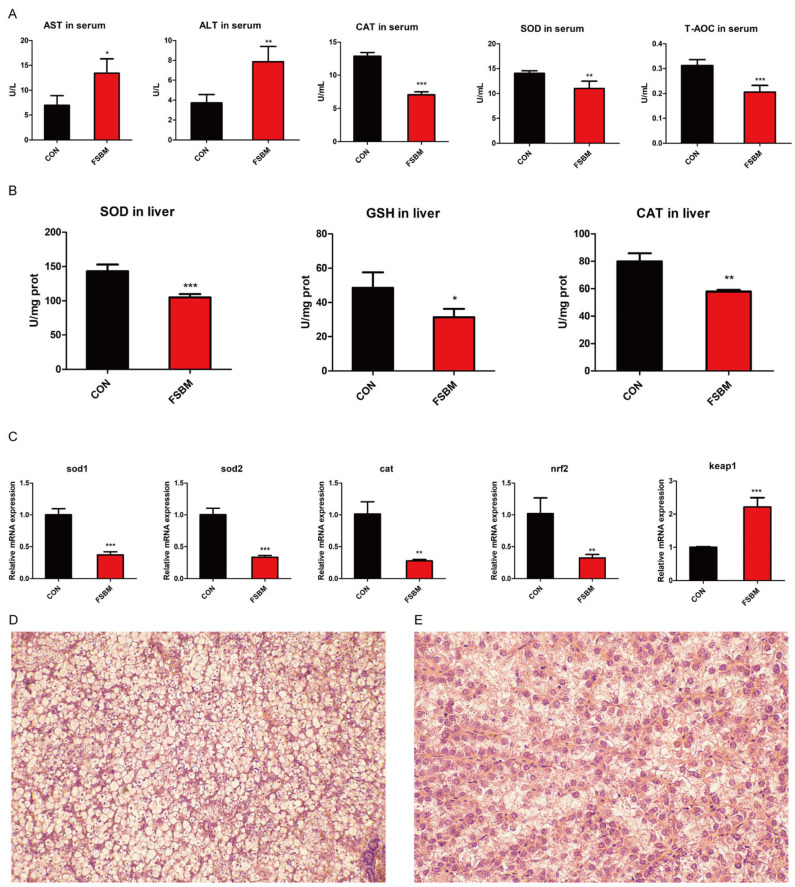
The effects of FSBM substitution on the hepatic antioxidant capacity of largemouth bass were examined (*n* = 4). (**A**) The effects of FSBM substitution on the activities of AST, ALT, CAT, SOD and T-AOC in serum were analyzed. (**B**) The effects of FSBM substitution on CAT and SOD activities and GSH contents in the liver were detected. (**C**) The effects of FSBM substitution on the mRNA expression of antioxidant enzymes and the Keap1-nrf2 system in the liver were analyzed. (**D**,**E**) H&E staining of liver sections from different diet groups ((**D**) control; (**E**): FSBM. Scale bar, 25 μm). The results are reported as means ± SD and were evaluated using independent *t*-tests (* *p* < 0.05, ** *p* < 0.01, *** *p* < 0.001).

Anti-nutritional factors (ANFs) in vegetable materials not only affect aquatic animal digestion and absorption, but also induce oxidative stress and thus cause liver damage [31]. In the present study, the antioxidant indices in liver were determined. FSBM substitution significantly enhanced the activities of AST and ALT in serum (*p* < 0.05; Figure 3A), which indicates that FSBM substitution induced liver damage. Concomitantly, the activities of CAT, SOD and T-AOC in serum were also decreased by FSBM substitution (*p* < 0.05; Figure 3A). Correspondingly, FSBM substitution decreased the activities of CAT and SOD and the contents of GSH in the liver (*p* < 0.05; Figure 3B). The qRT-PCR results showed that FSBM substitution decreased the mRNA expression of antioxidant enzymes (*sod1*, *sod2* and *cat*) (*p* < 0.05; Figure 3C). Moreover, FSBM substitution disturbed the balance in the Keap1-Nrf2 system by activating Keap1 and inhibiting Nrf2 (*p* < 0.05; Figure 3C). Oxidative stress is often accompanied by the occurrence of inflammation. The results showed that FSBM substitution significantly enhanced the mRNA expression of proinflammatory cytokine (*tnf-α*, *il-6* and *il-1β*) (*p* < 0.05; Supplementary Figure S1). H&E sections showed that FSBM substitution increased the nucleus of hepatocytes (Figure 3D,E), indicating structural damage to the liver.

### 3.4. Transcriptome Analysis in the Liver Indicated That FSBM Substitution Affected Glycine, Serine and Threonine Metabolism and Glutathione Metabolism

**Figure 4:**
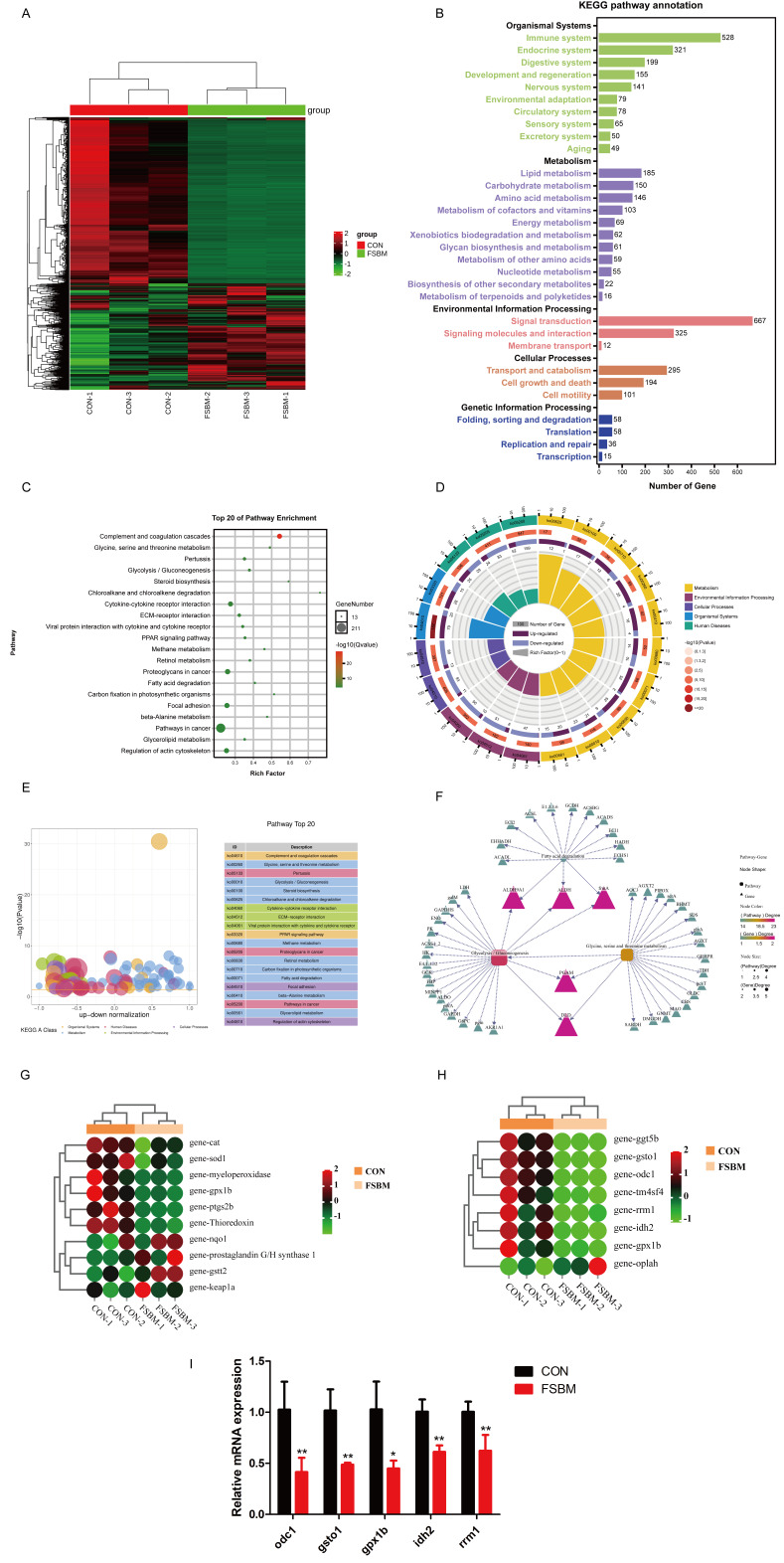
The transcriptomics data of hepatic DEGs in largemouth bass fed with different diets (*n* = 3). (**A**) Heat map. (**B**–**E**) KEGG pathway annotation. (**F**) Interaction network between metabolism-related genes and pathways. (**G**,**H**) Heat map of DEGs associated with antioxidant capacity and glutathione metabolism. (**I**) qPCR of genes involved in glutathione metabolism. For qPCR, the results are reported as means ± SD and were evaluated using independent *t*-tests (* *p* < 0.05, ** *p* < 0.01). DEGs, differentially expressed genes. See also Supplementary Figure S1.

To further analyze the mechanism by which FSBM substitution inhibited growth performance and induced liver oxidative stress, a transcriptome analysis in the liver was conducted. A total of 4230 differentially expressed genes (DEGs) were annotated, with 2538 downregulated and 1692 upregulated genes (Figure 4A). Gene Ontology (GO) function annotations indicated that most DEGs were enriched in a single-organism process, a cellular process and a metabolic process (biological process), cells, cell parts and membranes (cellular component), binding, catalytic activity and transporter activity (molecular function) (Supplementary Figure S2). To identify the active biological pathways in largemouth bass, DEGs were annotated using the Kyoto Encyclopedia of Genes and Genomes (KEGG). A total of 528 DEGs were enriched in the immune system and 667 DEGs were enriched in signal transduction (Figure 4B), which further indicates that FSBM substitution caused immune disorders. These immune disorders induced by external nutrients are often related to metabolic disorders in the liver. Hence, DEGs associated with metabolism attracted more attention. KEGG pathway annotation showed that 185 DEGs were enriched in lipid metabolism, 150 DEGs were enriched in carbohydrate metabolism and 146 DEGs were enriched in amino acid metabolism (Figure 4B). Specific pathway enrichments included glycine, serine and threonine metabolism, glycolysis/gluconeogenesis and fatty acid degradation (Figure 4C–E). To further demonstrate the relevance of the three major nutrient metabolisms in FSBM substitution, a KEGG advanced grid map was developed. *aldh9a1*, *aldh* and *frma* genes connected fatty acid degradation and glycolysis/gluconeogenesis. *pgam* and *dld* genes connected glycolysis/gluconeogenesis and glycine and serine and threonine metabolism (Figure 4F).

Further analysis of the changes in specific gene abundance revealed that FSBM substitution reduced the gene expression of antioxidant enzymes (Table 3; Figure 4G), which was validated by qPCR in some cases (Figure 3C). Considering that FSBM substitution affected glycine, serine and threonine metabolism and that glycine is a precursor to the synthesis of glutathione, an important antioxidant mediator, 14 DEGs associated with glutathione metabolism were also annotated (Table 4; Figure 4H), some of which (*odc1*, *gsto1*, *gpx1b*, *idh2* and *rrm1*) were validated via qPCR (Figure 4I). Taken together, these results indicated that a fluctuating glutathione metabolism might contribute to oxidative stress in the liver.

### 3.5. FSBM Substitution Changed the Metabolome Profile in the Liver

**Figure 5:**
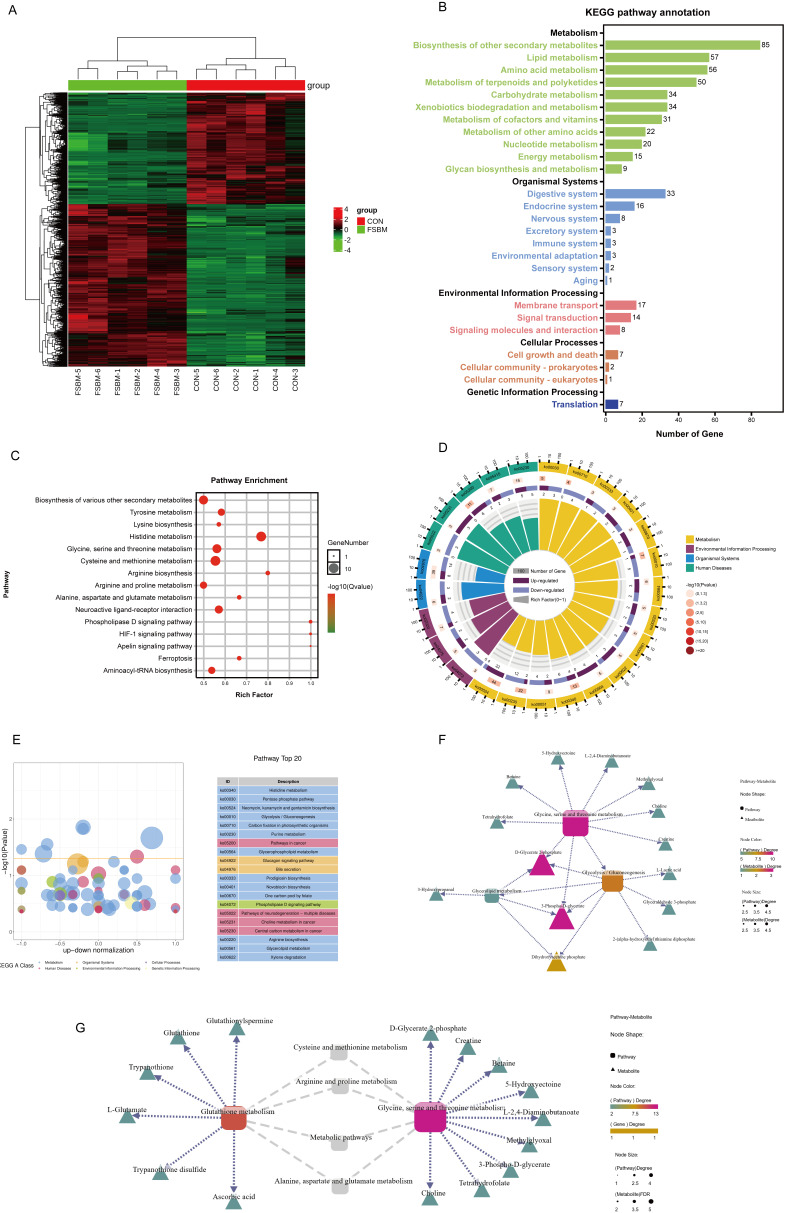
The DMs screened by metabolomic analysis in the liver of largemouth bass fed different diets (*n* = 6). (**A**) Heat map. (**B**–**E**) KEGG pathway annotation. (**F**) Interaction network between metabolism-related metabolites and pathways. (**G**) Interaction network between glutathione metabolism and glycine, serine and threonine metabolism. DMs, different metabolites.

Through a transcriptome analysis, it was elucidated that FSBM substitution affected glycine, serine and threonine metabolism, as well as glutathione metabolism. A metabolomics analysis was performed to more directly analyze the trends of changes in metabolite profiles. A total of 1994 different metabolites (DMs) were annotated, including 811 downregulated and 1183 upregulated different metabolites (Figure 5A). KEGG pathway annotation showed most DMs were enriched in metabolism, including biosynthesis of other secondary metabolites and lipid metabolism, especially amino acid metabolism (Figure 5B). Specific pathways were mainly histidine metabolism, glycine, serine and threonine metabolism, glycolysis/gluconeogenesis, glycerolipid metabolism and the phospholipase D signaling pathway (Figure 5C–E). Similar to the transcriptome analysis, KEGG advanced grid maps were created to connect the metabolism of three major nutrients. D-glycerate 2-phosphate and 3-phospho-D-glycerate connected glycine, serine and threonine metabolism, glycerolipid metabolism and glycolysis/gluconeogenesis. Dihydroxyacetone phosphate connected glycerolipid metabolism and glycolysis/gluconeogenesis (Figure 5F).

The DMs associated with glutathione metabolism were also annotated. Moreover, glutathione metabolism and glycine, serine and threonine metabolism were connected by other amino acid metabolisms, including cysteine and methionine metabolisms, arginine and proline metabolisms and alanine, aspartate and glutamate metabolisms (Figure 5G). Compared with the CON diet, FSBM substitution decreased ascorbic acid, L-glutamate and glutathione levels while increasing trypanothione, trypanothione disulfide and glutathionylspermine levels (Table 5). The trend in GSH was confirmed by the commercial kits (Figure 3B). Correspondingly, a metabolome analysis also found that FSBM substitution increased the contents of oxidized glutathione (Table 6). Overall, the metabolomics data indicate that FSBM substitution impaired glutathione metabolism, which is consistent with the transcriptome data.

### 3.6. Integrated Analysis of Transcriptomics and Metabolomics Data

**Figure 6:**
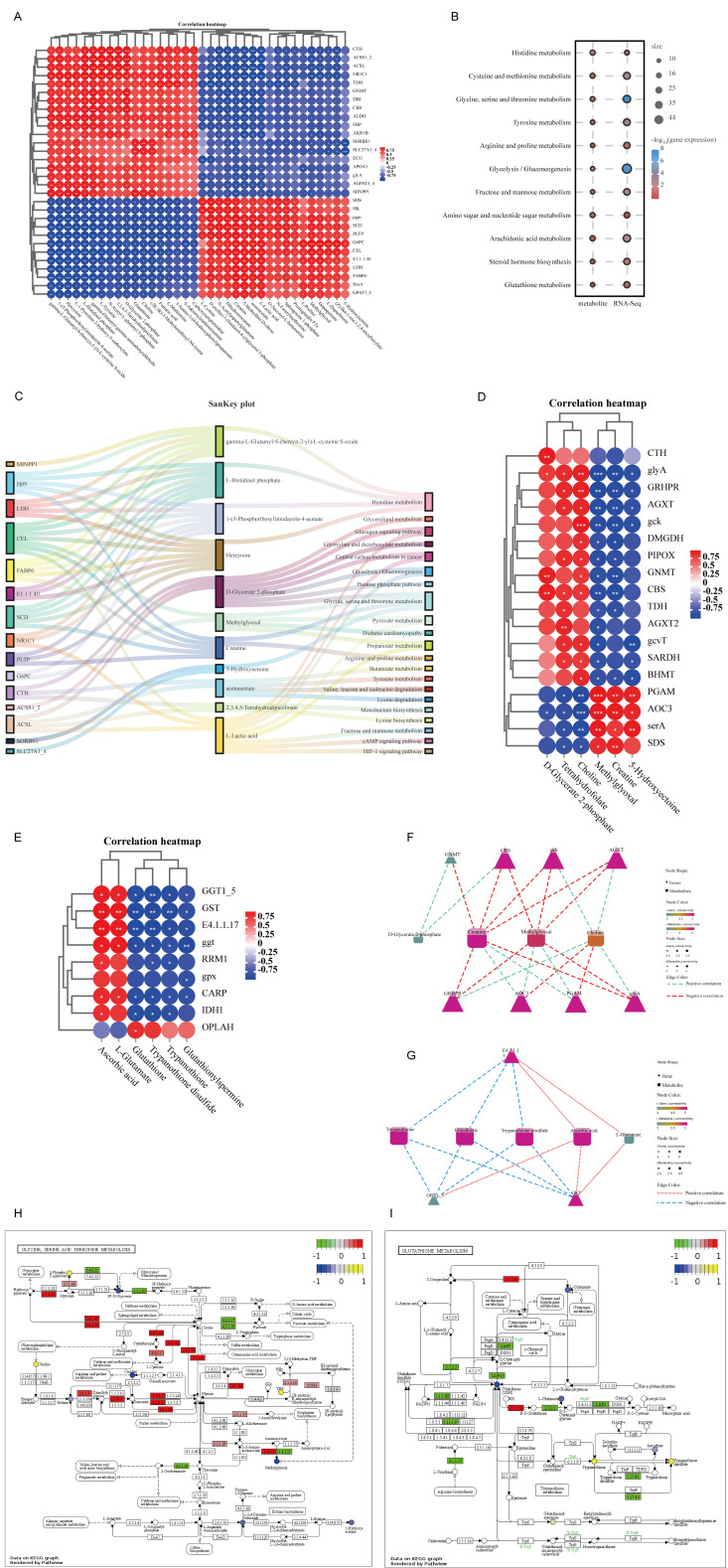
DEG–DM integrated analysis in the liver of largemouth bass fed different diets. (**A**) A correlation analysis between DEGs and DMs. (**B**) In KEGG pathways, transcriptome and metabolome pathways are annotated simultaneously. The metabolome is shown on the left, while the transcriptome is shown on the right. (**C**) Sankey plot of DEG–DM pathway correlation in the liver of largemouth bass. (**D**) Gene metabolite association heat map in glycine, serine and threonine metabolism. (**E**) Gene metabolite association heat map in glutathione metabolism. (**F**,**G**) The most significantly enriched DEGs and DMs interaction network diagram in glycine, serine and threonine metabolism as well as glutathione metabolism. (**H**,**I**) DEG and DM KEGG mapping results were enriched in glycine, serine and threonine metabolism as well as glutathione metabolism. The number of “*” in the circle represents the degree of correlation.

Both the transcriptomics and metabolomics data indicated that FSBM substitution affected glycine, serine and threonine metabolism, as well as glutathione metabolism. To better characterize DMs and explore the relationship between DMs and DEGs, an integrated analysis of transcriptomics and metabolomics data was conducted. A correlation analysis of DMs and DEGs related to three major nutrient metabolisms was performed (Figure 6A). Under FSBM substitution, histidine metabolism, glycine, serine and threonine metabolism, glycolysis/gluconeogenesis, arachidonic acid metabolism and glutathione metabolism were annotated in the common enrichment pathway of DEGs and DMs (Figure 6B). A Sankey plot associated DMs with DEGs mapped them to the relevant signaling pathways (Figure 6C). Further investigation revealed an association network consisting of 18 major genes and 6 DMs, with two different positive and negative correlation co-expression modules (Figure 6D). A directed grid graph analysis identified that there was a network of eight core genes (*gnmt*, *cbs*, *gck*, *agxt*, *grhpr*, *aoc3*, *pgam* and *glya*) and four metabolites (d-glycerate 2-phosphate, creatine, methylglyoxal and choline) in glycine, serine and threonine metabolism (Figure 6F). KEGG mapping of DEGs and DMs showed that FSBM substitution significantly affected glycine, serine and threonine metabolism (Figure 6H). Correspondingly, there was also an association network consisting of nine core genes and six DMs in glutathione metabolism (Figure 6E). *ggt1_5*, *gst* and E4.1.1.17 (*odc1*) genes connected trypanothione, glutathione, trypanothione disulfide, ascorbic acid and L-glutamate metabolites (Figure 6G). KEGG mapping of DEGs and DMs showed that glutathione metabolism was also significantly affected under FSBM substitution (Figure 6I).

## 4. Discussion

Aquaculture provides humans with many high-quality proteins and is an important guarantee of food security. However, the shortage of FM resources seriously limits the sustainable development of aquaculture. SBM is a crucial protein source in aquatic animal feed. Various biological technologies (enzymatic hydrolysis and fermentation) are used to improve its biological characteristics, making SBM more suitable for digestion and absorption by aquatic animals [32,33,34]. There are still many challenges in replacing FM with FSBM. The present study used a largemouth bass model to explore the mechanism of oxidative stress in the liver under a 50% substitution of FM by FSBM through a combined transcriptomics and metabolomics analysis system. The results indicated that a damaged glycine, serine and threonine metabolism, as well as glutathione metabolism, promoted the occurrence of oxidative stress, revealing the importance of non-essential amino acids in fish feed.

The development of FSBM focuses largely on the selection of bacteria. The FSBM used in the current study was fermented using *Bacillus subtilis*, which has been widely used in feed for aquatic animals, including *Scophthalmus maximus* [35], *Litopenaeus vannamei* [36], *Rana catesbiana* [37] and largemouth bass [21]. However, the ratio of FM substitution should be limited, as excessive substitution not only reduces animal growth but also induces oxidative stress, even after artificial essential amino acid supplementation, which was corroborated in the present study. Fermentation efficiently scavenges ANFs in FSBM; thus, the limiting factor may be the amino acid composition of the protein itself. The amino acid composition of feed often affects amino acid profiles in fish. In the present study, FSBM substitution had little effect on the free amino acid composition in the serum of largemouth bass, only affecting the contents of His, Phe, Asn and Orn. However, FSBM substitution significantly reduced the levels of free amino acids in muscle, including total EAAs, total NEAAs and total free amino acids, which is a little contradictory to previous studies. Results from largemouth bass and crabs indicated that FSBM substitution had a weak effect on the content of stable amino acids in muscle [38,39]. However, the above studies indicated that FSBM substitution reduced both the content of free and stable glycine in muscles. Further analysis revealed that FSBM reduced the expression of amino acid transporters (*lat1*, *y + lat2* and *y + lat1*) and a small peptide transporter (*pept2*). *Lat1* mediates the uptake of large neutral amino acids such as Phe, Tyr, His, Met, Trp, Val, Ile and Ala [40]. *Y + lat2* is responsible for the transport of cationic amino acids (such as Arg) and neutral amino acids (such as Leu, Glu and Ile) [41]. Previous research in animals found that a higher availability of EAAs boosted muscle *lat1* and *y + lat2* expression [42]. In the present study, excessive dietary FSBM decreased the availability of EAAs and inhibited *lat1* and *y + lat2* expression in muscle, which has been confirmed by Wang et al. [43] and Sheng et al. [44]. *Pept2* transports oligopeptides of two to four amino acids, with a preference for dipeptides, which can be activated by protein sources rich in small peptides [45]. However, high levels of FSBM inhibited the expression of *pept2*, which is consistent with previous results [46]. Overall, the decline in the expression of amino acid and small peptide transporters partly explains why FSBM substitution affected specific amino acid contents.

Another factor restricting FSBM replacement is that an excessive substitution ratio reduces the immunity of aquatic animals and induces oxidative stress. In the present study, FSBM substitution significantly enhanced the activities of ALT and AST. Correspondingly, FSBM substitution inhibited the activities of antioxidant enzymes in the serum and liver, together with the mRNA expression of antioxidant enzymes. Similar results were also shown in *Macrobrachium nipponense* [47] and *Lateolabrax japonicus* [48]. Intracellular oxidative and antioxidant systems are in dynamic balance. Among them, Keap1-Nrf2 is a critical antioxidant signaling pathway. Keap1 mediates the degradation of Nrf2 by proteasomes with a physiological status. Hence, Nrf2 is kept in a low expression state. Under stress, Nrf2 dissociates from Keap1 and enters the nucleus to initiate transcription of antioxidant enzymes [49]. In the present study, FSBM substitution activated Keap1 and inhibited Nrf2. Hence, the antioxidant systems were disrupted, resulting in damage to the liver structure. In order to gain a more comprehensive understanding of the transcriptional changes caused by FSBM substitution, a transcriptome analysis was conducted. In addition, DEGs related to antioxidant activity were annotated, demonstrating the accuracy of transcriptome sequencing. Due to the fact that diseases caused by nutrient availability are mainly attributed to metabolic disorders in the body, we have focused on DEGs related to the metabolism of three major nutrients, namely glycine, serine and threonine metabolism. Glycine is one of the most important components of intracellular antioxidant GSH. GSH mediates glutathione peroxidase (GPX) to exert antioxidant enzyme activity, catalyzing the conversion of H_2_O_2_ into H_2_O and aiding the transition from reducing GSH to oxidizing glutathione (GSSG) during this process [50]. *gnmt*, *dmgdh* and *gcvh* genes were all upregulated by FSBM substitution, which mediates glycine degradation [51]. Hence, intracellular GSH levels decreased in the FSBM group. In addition, DEGs associated with GSH metabolism were also annotated. *ggt5b* and *gsto1* genes are responsible for GSH synthesis [52] and were both inhibited by FSBM substitution. Correspondingly, *gpx1b* decreased in the FSBM group. Overall, these data on the transcriptional level determined that disorders of glycine, serine and threonine metabolism and glutathione metabolism reduced the cellular antioxidant capacity.

The results of simple transcriptomics are partial, since there is a long biochemical process from genes to glutathione compounds that exert antioxidant functions. Therefore, we conducted a metabolomics analysis to further determine the role of glutathione metabolism in oxidative stress induced by FSBM. The KEGG enrichment results indicated that most DMs were enriched in metabolic processes. Similar to the transcriptomics analysis, glycine, serine and threonine metabolism and glutathione metabolic pathways were enriched. Betaine, chemically known as N, N, N-trimethylglycine, is often used as an attractant in aquatic feeds and has been shown to have strong antioxidant effects [53]. In the present study, the content of betaine was reduced by FSBM substitution. Creatine, which is synthesized from arginine, glycine and methionine, provides energy for biological activities and exhibits some antioxidant activities [54], and its content was decreased in the FSBM group. The KEGG advanced grid graph showed that glycine, serine and threonine metabolism is linked to glutathione metabolism by other metabolic pathways. Hence, metabolic disorders of glycine, serine and threonine are bound to affect glutathione metabolism. A metabolome analysis demonstrated that glutathione contents were reduced, while the GSSG contents were increased. The decrease in the ratio of GSH/GSSG also indicated the impairment of liver antioxidant capacity [55]. In addition, FSBM substitution also decreased the contents of L-glutamate, another amino acid that makes up glutathione. Taken together, these results indicated that FSBM substitution impaired glutathione metabolism, partially by reducing the contents of glycine and glutamate. Both transcriptomics and metabolomics data have demonstrated that FSBM substitution may cause oxidative stress by affecting glycine, serine and threonine metabolism, as well as glutathione metabolism. In order to find more accurate targets, DEGs and DMs involved in the two pathways were jointly analyzed. For glycine, serine and threonine metabolism, there are four key metabolites, including creatine, choline, D-glycate 2-phosphate and methylglyoxal. The intracellular metabolism of creatine and choline is closely related to glycine. Emerging evidence has shown that dietary creatine and choline supplementation alleviate oxidative stress in aquaculture [54,56]. Along with the results in the present study, the necessity of creatine and choline supplementation was highlighted in improving plant-based protein utilization. In addition, genes involved in glycine metabolism, including *gnmt*, *glya* and *agxt*, were core targets to relieve oxidative stress [51]. For glutathione metabolism, three key genes (*odc1*, *ggt* and *gst*) connected trypanothione, glutathione, trypanothione disulfide, ascorbic acid and L-glutamate. Among them, glutathione and ascorbic acid are both efficient antioxidants. The KEGG mapping results of DEGs and DMs further intuitively demonstrated the regulatory effect of FSBM on glycine, serine and threonine metabolism as well as glutathione metabolism.

In conclusion, the present study established a liver oxidative stress model in largemouth bass using 50% FSBM substitution and utilized transcriptome and metabolomic analyses to elucidate that impaired glycine, serine and threonine metabolism, as well as glutathione metabolism, exacerbated liver oxidative stress. Key genes (*odc1*, *gnmt*, *agxt*, *ggt* and *gst*) and core metabolites (glutathione, glutamate and glycine) should be evaluated to improve the availability of FSBM. The importance of non-essential amino acids in the adaptation process of plant-based protein sources is thus emphasized. Considering that glycine not only acts as an antioxidant, but also promotes muscle development and exerts anti-inflammatory effects, it is clear that dietary glycine supplementation has a significant effect on improving the utilization of plant-based protein sources.

## 5. Study Limitations

Our in vivo experiment demonstrated that 50% substitution of FM with FSBM induces oxidative stress by disturbing glycine and glutathione metabolism in largemouth bass, mainly according to the decreased activities and expressions of antioxidant enzymes. The transcriptomics and metabolomics data also validated the regulatory network during FSBM substitution. However, we did not clarify the temporal dynamics of gene expression and the potential for compensatory mechanisms. Hence, this manuscript is a preliminary work. Long-term studies, including in vitro experiments involving RNA knock down and gene expression kinetic analyses, are necessary.

## Figures and Tables

**Table 1:** Formulation and chemical proximate composition of the experimental diets (% dry matter).

Ingredients	Diets ^a^	
	CON	FSBM
Fish meal	62	31
Fermented soybean meal ^b^	0	40.25
Alpha-starch	10	10
Microcrystalline cellulose	12.51	3.03
Fish oil	3	4
Soybean oil	4	3.23
Soybean lecithin	1	1
Choline	0.2	0.2
Monocalcium phosphate	1	1
Sodium alginate	1	1
Vitamin C	1	1
Mineral mix ^c^	3	3
Vitamin premix ^d^	0.05	0.05
Mold inhibitor ^e^	0.2	0.2
Ethoxyquin	0.01	0.01
L-lysine	0	0.58
L-methionine	0	0.45
Alanine	1.03	0
Total	100	100
Proximate analysis		
Crude protein	46.74	48.29
Crude lipid	11.9	11.67

^a^ CON, control group; FSBM, group in which 50% FM was substituted with fermented soybean meal. ^b^ FSBM was fermented using *Bacillus subtilis*. The amino acid composition is presented in Supplementary Table S1. ^c^ Mineral premix (g/kg): the mineral premix formula was taken from the article published by Li et al. [26]. The details are as follows: CoCl·6H_2_O, 0.86; MgSO_4_·7H_2_O, 586.67; ZnSO_4_·7H_2_O, 18.09; NaCl, 363.88; FeSO_4_·7H_2_O, 22.22; KI, 0.67; AlCl_3_·6H_2_O, 0.67; Na_2_SeO_3_, 0.06; CuSO_4_·5H_2_O, 2.22; MnSO_4_, 4.67. ^d^ Vitamin premix (g/kg): the vitamin premix formula was taken from a previously published article [26]. The details are as follows: Inositol, 15.00; vitamin A, 2.31; vitamin B1, 3.26; vitamin B2, 3.00; vitamin B5, 10.87; vitamin B6, 3.04; niacin, 14.00; biotin, 0.15; folic acid, 0.60; vitamin B12, 0.02; vitamin D3, 2.02; vitamin E, 20.00; vitamin K3, 1.20; cellulose, 924.53. ^e^ Mold inhibitor contained 50% calcium propionic acid and 50% fumaric acid.

**Table 2:** Amino acid composition of experimental diets (% dry weight).

Amino Acid	Diets	
	CON	FSBM
Essential amino acids (EAAs)		
Arginine	2.98	2.89
Histidine	0.98	1.03
Isoleucine	2.04	1.92
Lysine	3.39	2.95
Methionine	1.26	1.1
Phenylalanine	1.90	2.03
Threonine	2.10	1.76
Valine	2.17	1.95
Leucine	3.58	3.23
Non-essential amino acids (EAAs)		
Alanine	2.98	3.12
Aspartate	4.50	4.66
Cysteine	0.17	0.26
Glycine	3.06	2.37
Glutamate	6.52	7.76
Proline	2.38	2.38
Serine	2.22	2.09
Tyrosine	1.29	1.39
Total	43.53	42.88

Tryptophan was unable to be identified due to destruction during acid hydrolysis.

**Table 3:** Antioxidant-related genes in response to FSBM.

Gene Name	KEGG Annotation	Log_2_FC	Regulated	KEGG Pathway
*cat*	K03781	−1.38	down	Peroxisome
*nqo1*	K00355	1.36	up	Ubiquinone and other terpenoid quinones Biosynthesis
*mpo*	K10789	−2.00	down	Phagosome
*gpx1b*	K00432	−1.68	down	Glutathione metabolism
*ptgs2b*	K11987	−2.15	down	Arachidonic acid metabolism
*trx*	K03671	−1.58	down	NOD-like receptor signaling pathway
*ptgs1*	K00509	1.67	up	Arachidonic acid metabolism
*keap1a*	K10456	1.31	up	Ubiquitin-mediated proteolysis
*sod1*	K04565	−1.49	down	Peroxisome
*gstt2*	K00799	1.32	up	Glutathione metabolism

**Table 4:** Glutathione-metabolism-related genes in response to FSBM.

KEGG Annotation	Gene Name	Log_2_FC	Regulated
Glutathione metabolism	*ggt5b*	−1.59	down
	*tm4sf4*	−1.53	down
	*gsta.1*	1.41	up
	*gstt3*	1.84	up
	*oplah*	2.01	up
	*gsto1*	−1.40	down
	*gpx3*	2.01	up
	*odc1*	−2.23	down
	*idh2-like*	−2.26	down
	*rrm1*	−1.08	down
	*gstt3-like*	1.95	up
	*gpx1b*	−1.68	down
	*idh2*	−1.54	down
	*gstt2*	1.32	up

*ggt5b*, glutathione hydrolase; *tm4sf4*, transmembrane 4 L6 family member 4; *gsta.1*, glutathione S-transferase A1; *gstt3*, glutathione S-transferase theta-3; *oplah*, 5-oxoprolinase; *gsto1*, glutathione S-transferase omega-1; *gpx3*, glutathione peroxidase 3; *odc1*, ornithine decarboxylase 1; *idh2-like*, isocitrate dehydrogenase (NADP), mitochondrial; *rrm1*, ribonucleoside-diphosphate reductase large subunit 1; *gstt3-like*; glutathione S-transferase theta-3-like; *gpx1b*, glutathione peroxidase 1b; *idh2*, isocitrate dehydrogenase (NADP), mitochondrial; *gstt2*, glutathione S-transferase theta-2.

**Table 5:** Glycine, serine and threonine metabolism-related metabolites in response to FSBM.

KEGG Annotation	Metabolite Name	Log_2_FC	Regulated
Glycine, serine and threonine metabolism	D-Glycerate 2-phosphate	4.24	Up
	Methylglyoxal	−3.11	Down
	Creatine	−0.89	Down
	Betaine	−0.36	Down
	3-Phospho-D-glycerate	−0.61	Down
	5-Hydroxyectoine	−0.60	Down
	L-2,4-Diaminobutanoate	−0.57	Down
	Tetrahydrofolate Choline	0.94 0.65	Up Up

**Table 6:** Glutathione-metabolism-related metabolites in response to FSBM.

KEGG Annotation	Metabolite Name	Log_2_FC	Regulated
Glutathione metabolism	Ascorbic acid	−0.31	Down
	L-glutamate	−0.50	Down
	Glutathione	−2.61	Down
	Trypanothione	1.23	Up
	Glutathione, oxidized	1.04	Up
	Trypanothione disulfide	4.26	Up
	Glutathionylspermine	0.36	Up

## Data Availability

All relevant data are within the manuscript and the Supplementary Materials. Any additional information required to re-analyze the data reported in this paper is available from the lead author upon request.

## Supplementary Materials

The following supporting information can be downloaded at: https://www.mdpi.com/article/10.3390/antiox12122096/s1, Figure S1: Effects of FSBM substitution on inflammatory response in liver of largemouth bass (*n* = 4); Figure S2: GO functional classification of the differentially expressed proteins after treatment with different diets (*n* = 3); Table S1: Amino acid composition of the fermented soybean meal (% dry weight); Table S2: Primer sequences of genes used for qRT-PCR. Table S3: The normality and homoscedasticity analysis of Figure 1; Table S4: The normality and homoscedasticity analysis of Figure 2; Table S5: The normality and homoscedasticity analysis of Figure 3; Table S6: The normality and homoscedasticity analysis of Figure 4.
