# Supplementary material for: Excessive Substitution of Fish Meal with Fermented Soybean Meal Induces Oxidative Stress by Impairing Glutathione Metabolism in Largemouth Bass (Micropterus salmoides)
Source: Antioxidants (Basel). 2023 Dec 11;12(12):2096. doi: 10.3390/antiox12122096 (PMC10740881; doi:10.3390/antiox12122096)
Supplement: Supplementary file 1 [file antioxidants-12-02096-s001.zip › antioxidants-2706130-supplementary.pdf]

Supplementary Table S1. Amino acid composition of the fermented soybean meal (% dry weight)

Amino acid	
Essential amino acids (EAAs)	
Histidine	1.13
Threonine	1.52
Arginine	3.10
valine	1.79
Methionine	0.45
Phenylalanine	2.44
Isoleucine	1.87
leucine	3.22
lysine	2.60
Non-essential amino acids (EAAs)	
Aspartate	4.04
Glutamate	9.21
Serine	1.98
Glycine	1.63
Alanine	1.99
Tyrosine	1.54
Proline	2.56
Total	41.07

Tryptophan could not be determined because of degradation during acid hydrolysis

Supplementary Table S2. Primer sequences of genes used for qRT-PCR.

Sequence Name	Forward 5'-3'	Reverse 5'-3'	Accession number
gapdh	ACTGTCACTCCTCCATCTT	CACGGTTGCTGTATCCAA	XM_038711150.1
lat1	CCAAAGCACGACAGACCTACA	ACCAACCTGGCATATTTCACC	XM_038706332.1
y+lat2	TCTGCCCTCTTCTCCTATTCC	TGTCACTGCCACTGCATCACT	XM_038700945.1
y+lat1	TGGTGTAATGAACTGGACGAT	GAGCCATGATGCAATTAAAGA	XM_038731565.1
bcat2	CAGAACGAGGCGATAAAGAA	CATTGGTCCAGTAGATGAAGAG	XM_038706627.1
pept2	CACTGGTGGAGGTGAATGTTGT	TTTAGGAGGGTCCTGAAGGTAT	XM_038721077.1
odc1	TGCACTGATCCCGAGACCTA	GGGCAGGGTTGATTACTGCT	XM_038728782.1
gsto1	CGCCCAGAGAGCCAGATTAG	CAGTCCTGAGACATCCTCGC	XM_038737564.1
idh2	TATGACCTGGGTCTGCCGTA	TTGCCATCAAGTTTGCCACG	XM_038705628.1
rrm1	CGGCACTCTCCCATGATCTC	CTGTGCAAAGGTTGCTGCAT	XM_038694259.1
tnf-α	CTTCGTCTACAGCCAGGCATCG	TTTGGCACACCGACCTCACC	XM_038723994.1
il-6	GACTGGAGTGGCGGAAAGTGGAGG	TTTCATCTTCTACAAACGCAGACAACGG	XM_038711438.1
il-1β	CGTGACTGACAGCAAAAAGAGG	GATGCCCAGAGCCACAGTTC	XM_038733429.1
sod1	TTATTTTGAGCAGGAGGG	TTCTTGTTGTGGGGATTG	XM_038708943.1
sod2	GGTCTCATTCCCCTTCTT	TCGCTCACATTCTCCCAG	XM_038727054.1
cat	TGAATGGCTATGGCTCTC	AATCTGGGTTGGTGGAAG	XM_038704976.1
gpx1b	CTCCTCAACCAGGCAAAC	ATACCCCCCTCACAACAA	XM_038697919.1
keap1	AGACGGCAGGAGATGTTGT	CATGGCTCTGAAGTAGGGG	XM_038728593.1
nrf2	CACCAAAGACAAGCGTAAG	GAAATCATCAACAGGCAGA	XM_038720536.1

Gapdh, Glyceraldehyde-3-phosphate dehydrogenase; *lat1*, Solute carrier family 7 member 5; *y+lat2*, Solute carrier family 7 member 6; *y+lat1*, Solute carrier family 7 member 7; *bcat2*, Branched-chain-amino-acid aminotransferase, mitochondrial; pept2, Solute carrier family 15 member 2; *odc1*, Ornithine decarboxylase 1; *gsto1*, Glutathione S-transferase omega-1; *idh2*, Isocitrate dehydrogenase [NADP], mitochondrial; *rrm1*, Ribonucleoside-diphosphate reductase large subunit 1; *tnf-α*, tumor necrosis factor-α; *il-6*, Interleukin 6; *il-1β*, Interleukin 1β; *sod1*, Superoxide dismutase [Cu-Zn]; *sod2*, Superoxide dismutase [Mn], mitochondrial; *cat*, Catalase; *gpx1b*, Glutathione peroxidase 1b; *keap1*, Kelch-like ECH-associated protein 1; *nrf2*, Nuclear factor erythroid 2-related factor 2

Supplementary Table S3. The normality and homoscedasticity analysis of Figure 1

Indexes	P value of normality[1]	P value of homoscedasticity[2]	P value[3]
FBW	0.156/0.415	0.797	0.002
WG	0.250/0.283	0.860	0.017
SGR	0.211/0.274	0.919	0.008
FER	0.622/0.344	0.446	0.005
HSI	0.849/0.587	0.166	0.007
VSI	0.430/0.577	0.068	0.042
SR	0.156/0.126	1.000	0.519

[1] P > 0.05 indicates that the data conforms to a normal distribution;

[2] P > 0.05 represents the variance of the data;

[3] P > 0.05 indicates no significant difference in data.

Supplementary Table S4. The normality and homoscedasticity analysis of Figure 2

Indexes	P value of normality[1]	P value of homoscedasticity[2]	P value[3]
Serum-his	0.338/0.439	0.050	0.013
Serum-thr	0.061/0.369	0.457	0.144
Serum-arg	0.835/0.497	0.347	0.903
Serum-val	0.715/0.454	0.226	0.355
Serum-met	0.589/0.597	0.131	0.910
Serum-trp	0.627/0.783	0.129	0.557
Serum-phe	0.460/0.722	0.120	0.032
Serum-ile	0.852/0.208	0.217	0.360
Serum-leu	0.644/0.098	0.224	0.302
Serum-lys	0.272/0.491	0.057	0.270
Serum-TEAAs	0.983/0.106	0.517	0.948
Serum-asp	0.152/0.703	0.173	0.130
Serum-glu	0.981/0.481	0.652	0.920
Serum-asn	0.726/0.891	0.682	0.014
Serum-ser	0.884/0.063	0.738	0.618
Serum-gln	0.441/0.670	0.166	0.243
Serum-gly	0.066/0.384	0.420	0.007
Serum-cit	0.323/0.703	0.541	0.195
Serum-ala	0.780/0.985	0.186	0.766
Serum-tau	0.849/0.453	0.131	0.940
Serum-tyr	0.610/0.202	0.221	0.152
Serum-orn	0.646/0.714	0.064	0.007
Serum-pro	0.188/0.118	0.099	0.419
Serum-TNEAAs	0185/0.065	0.233	0.611
Serum-TAAs	0.125/0.075	0.242	0.684
Muscle-his	0.926/0.073	0.823	0.012
Muscle-thr	0.518/0.406	0.776	0.008
Muscle-arg	0.994/0.400	0.182	0.001
Muscle-val	0.210/0.215	0.585	0.349
Muscle-met	0.949/0.054	0.377	0.769
Muscle-phe	0.533/0.884	0.091	0.000
Muscle-ile	0.587/0.908	0.048	0.061
Muscle-leu	0.996/0.246	0.219	0.045
Muscle-lys	0.966/0.900	0.167	0.004
Muscle-TEAAs	0.370/0.305	0.045	0.033
Muscle-asp	0.134/0.630	0.183	0.121
Muscle-glu	0.724/0.930	0.132	0.012
Muscle-ser	0.216/0.952	0.033	0.028
Muscle-gly	0.186/0.541	0.067	0.002
Muscle-ala	0.066/0.909	0.022	0.010

Muscle-tyr	0.537/0.659	0.252	0.004
Muscle-pro	0.285/0.725	0.154	0.000
Muscle-TNEAAs	0.869/0.778	0.305	0.000
Muscle-TAAs	0.579/0.579	0.345	0.000
Lat1	0.291/0.723	0.086	0.002
Y+lat2	0.143/0.301	0.000	0.026
Y+lat1	0.294/0.589	0.093	0.000
Pept2	0.985/0.995	0.060	0.000
Bcat2	0.191/0.353	0.041	0.022

[1] $P > 0.05$ indicates that the data conforms to a normal distribution;

[2] $P > 0.05$ represents the variance of the data;

[3] $P > 0.05$ indicates no significant difference in data.

Supplementary Table S5. The normality and homoscedasticity analysis of Figure 3

Indexes	P value of normality[1]	P value of homoscedasticity[2]	P value[3]
AST	0.343/0.201	0.510	0.010
ALT	0.455/0.946	0.395	0.003
Serum CAT	0.420/0.935	0.413	0.000
Serum SOD	0.998/0.497	0.213	0.007
Serum T-AOC	0.443/0.615	0.792	0.001
Liver SOD	0.480/0.233	0.133	0.000
Liver GSH	0.065/0.977	0.260	0.017
Liver CAT	0.144/0.978	0.000	0.004
Sod1 mRNA	0.167/0.923	0.303	0.000
Sod2 mRNA	0.597/0.805	0.007	0.001
cat mRNA	0.976/0.647	0.026	0.004
Nrf2 mRNA	0.600/0.828	0.137	0.002
Keap1 mRNA	0.455/0.280	0.061	0.000

[1] $P > 0.05$ indicates that the data conforms to a normal distribution;

[2] $P > 0.05$ represents the variance of the data;

[3] $P > 0.05$ indicates no significant difference in data.

Supplementary Table S6. The normality and homoscedasticity analysis of Figure 4

Indexes	P value of normality[1]	P value of homoscedasticity[2]	P value[3]
Odc1	0.761/0.149	0.388	0.007
Gsto1	0.703/0.219	0.092	0.002
Gpx1b	0.198/0.562	0.000	0.020
Idh2	0.812/0.468	0.355	0.001
Rrm1	0.438/0.626	0.538	0.006

[1] P > 0.05 indicate0.436/0.626s that the data conforms to a normal distribution;

[2] P > 0.05 represents the variance of the data;

[3] P > 0.05 indicates no significant difference in data.

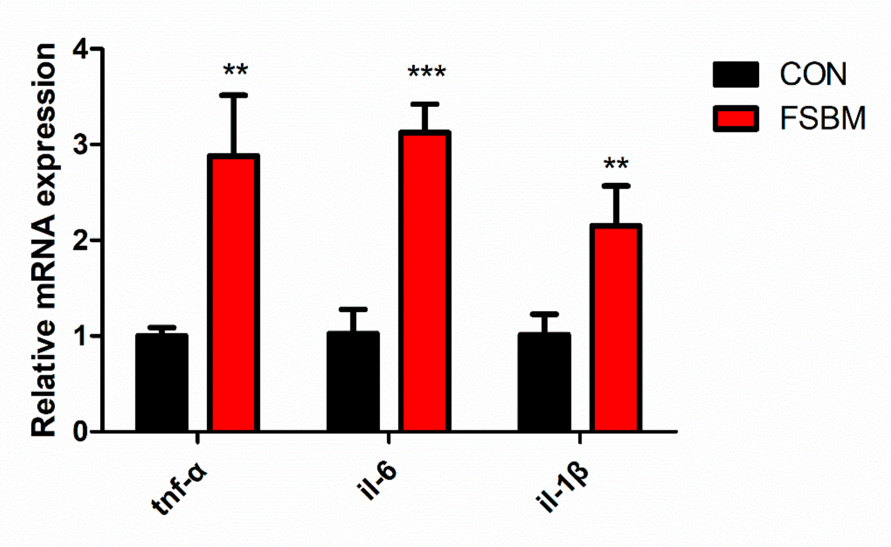

Supplementary Figure S1 Effects of FSBM substitution on inflammatory response in liver of largemouth bass (n = 4). The results are presented as the mean ± SD and were analyzed using independent t-tests (*P <0.05, **P <0.01, ***P <0.001).

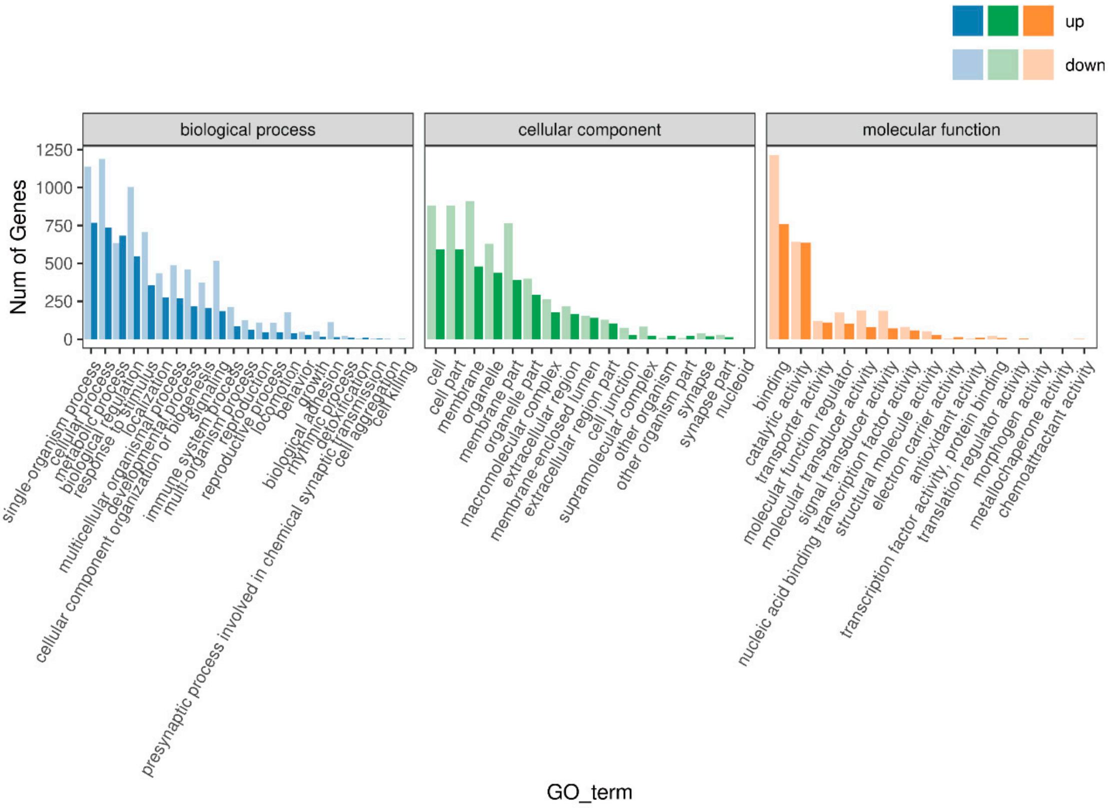

Supplementary Figure S2 GO functional classification of the differentially expressed proteins after treatment with different diets ($n = 3$). Related to Figure 3.
